# A plastic bezoar causing bowel obstruction: A case of table cover ingestion

**DOI:** 10.1016/j.ijscr.2024.109506

**Published:** 2024-03-11

**Authors:** Noran Sultan, Hanin Attar, Hatem Sembawa, Hind Alharthi

**Affiliations:** a General Surgery Department, King Abdulaziz Hospital, Makkah, Saudi Arabia; b General Surgery Department, Alnoor Specialist Hospital, Makkah, Saudi Arabia; c General Surgery Department, Umm Al-Qura University, Makkah, Saudi Arabia

**Keywords:** Plastic bezoar, Bezoar, Small bowel obstruction, Foreign body, Acute abdomen

## Abstract

**Introduction and importance:**

A bezoar is an indigestible food or other material within the gastrointestinal tract. It can be ingested intentionally or accidentally. The small bowel bezoar prevalence ranges between 0.4 % and 4 %, and the prevalence is less than 0.5 % for gastric bezoars. There are different types of bezoars, but the mention of a plastic bezoar rarely appears in the literature. To our knowledge, this is the first reported case of a plastic bezoar in the Kingdom of Saudi Arabia.

**Case presentation:**

A 58-year-old woman was admitted for acute kidney injury, and while working her up, it was discovered that she had a possible foreign body on computerized tomography scan. As a result, she underwent exploratory laparotomy with the findings of plastic foreign objects identified 90 cm from the ileocecal valve and other objects identified in the stomach.

**Clinical discussion:**

The impaction of these materials often occurs in narrow areas such as the lower esophagus, duodenum, ileocecal valve or even the anus. In this unique case, two points of impaction were noted: the first was in the small bowel and the second point in the stomach. The approach to such cases could be Endoscopic versus surgical or even chemical dissolution as a choice of treatment is dependent on multiple factors.

**Conclusion:**

The approach to these cases is multidisciplinary and depends on the availability of services and resources at the treating hospital. Reporting such cases helps in managing challenging situations. Additionally, a psychiatric assessment is a crucial step.

## Highlights

• It’s an underreported case in the literature
• We need to gather cases and put a preliminary approach in such conditions
• Early identification and management can limit dreadful complications specially in elderly patients.
• Such cases can enhance surgeons knowledge, awareness and operative skills.
• To spread mental health awareness through the community

## 1 Introduction

Bezoars are indigestible foods or other materials within the GIT. Bezoars can be ingested intentionally or accidentally [[1], [2], [3], [4]]. Most bezoars go unnoticed and are asymptomatic. The incidence of bowel obstruction from a bezoar is exceptionally low, Thus, it is an uncommon manifestation of small bowel obstruction (SBO), and the prevalence of small bowel bezoars ranges between 0.4 % and 4 %, while that of gastric bezoars is less than 0.5 % [1,4,6]. There are different types of bezoars, such as trichobezoars, phytobezoars, medicines such as cholestyramine and antacids (pharmacobezoars), lactobezoars, metal bezoars, and sand bezoars, but the mention of a plastic bezoar rarely appears in the literature [[2], [3], [4]]. It can present with a variety of symptoms and signs according to the location of the bezoar [9,10]. In our case, we present a patient who swallowed pieces of a plastic table cover and presented with an SBO, which is a scarce incident. To our knowledge, this is the first reported case of a plastic bezoar in the Kingdom of Saudi Arabia (KSA).

## 2 Methodology

The description of the case follows the 2023 SCARE Checklist Guidelines [12].

## 3 Case presentation

A 58-year-old woman was admitted due to acute kidney injury (AKI) under the care of the internal medicine department, their treatment plan was based on resuscitation with intravenous isotonic fluids and monitoring the urine output in response to the fluid replacement.

**Fig. 1:**
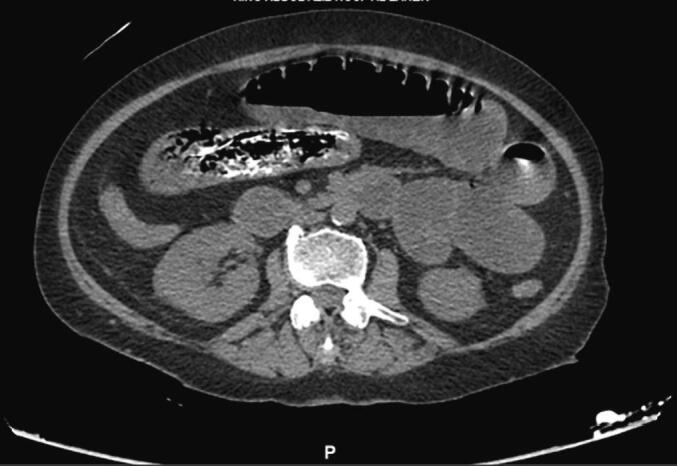
Foreign material seen within the small bowel (jejunum).

**Fig. 2:**
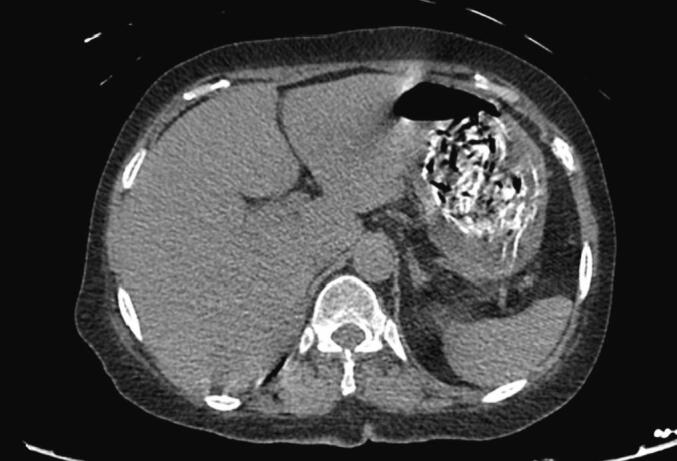
Foreign material seen within the stomach.

Five days after admission, a general surgery consultation was requested because of a plain computerized tomography (CT) scan of the kidneys, ureters and bladder (KUB), which revealed that the stomach and small bowel loops, likely jejunal loops, were filled with heterogeneous hyperdense material. The proximal small bowel loops were dilated with multiple air-fluid levels. A transition zone was observed in the small bowel in the pelvis. The large bowel was collapsed. The solid abdominal organs appeared unremarkable on nonenhanced CT. Both kidneys had a normal size, shape and parenchymal density with no stones or hydronephrosis. The urinary bladder was partially distended with a Foley catheter in situ. No free fluid or free air was observed. The scanned part of the chest appeared unremarkable. The diagnosis of a bezoar causing SBO was established (Fig. 1, Fig. 2).

The patient was not known to have any medical illness except for being mentally ill. In addition, there was no history of any previous surgeries or drug abuse and no previous similar complaint. The vague abdominal pain started two days prior to presentation to the hospital, it was not radiating or shifting and started two days prior to presentation. For the previous two days, the patient had a decrease in oral intake, which was not associated with nausea or vomiting. The patient had a positive minimal amount of loose stool. Regarding vital signs, the heart rate was 110/bpm, blood pressure was 110/80 mmHg, respiratory rate was 13/bpm, temperature was 37 degrees Celsius and oxygen saturation was 99 % on room air.

On examination, the patient was conscious but disoriented. The abdomen was distended, the patient was guarding her abdomen all over and she had exaggerated bowel sounds.

The initial nasogastric tube (NGT) output was 500 cc and brown in color. Digital rectal examination showed only loose stool. The urine output was minimal and concentrated. The decision was made to undergo emergency exploratory laparotomy with the impression of small intestinal obstruction due to a foreign body. Consent was obtained. The laboratory investigation showed a white blood cell count of 12,500/μL, a serum creatinine level of 492 mmol/L, a blood urea nitrogen (BUN) level of 40 mmol/L, a serum potassium level of 5 mmol/L and a serum sodium level of 125 mmol/L. Resuscitation with intravenous isotonic solutions, anti-hyperkalemia measures and renal doses of tazobactam were started (this is the pre-operative patient optimization that was done).

**Fig. 3:**
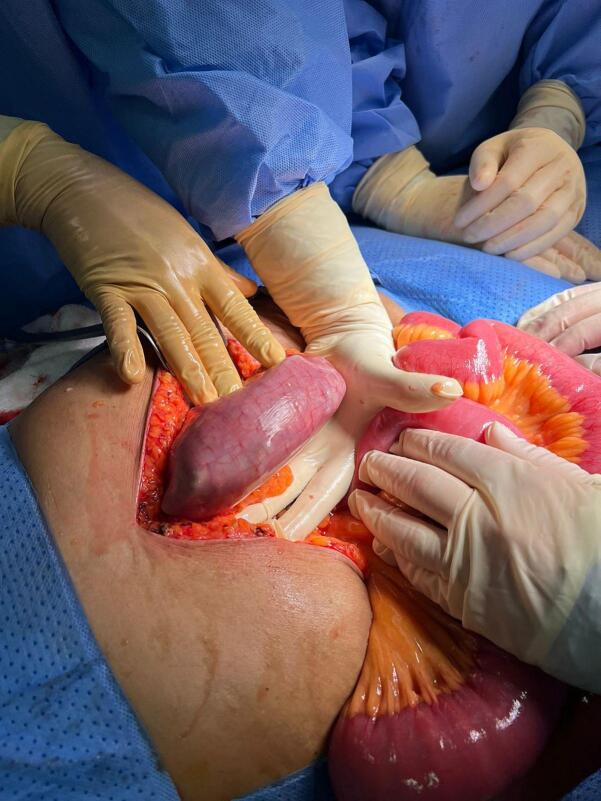
A jejunal foreign body.

**Fig. 4:**
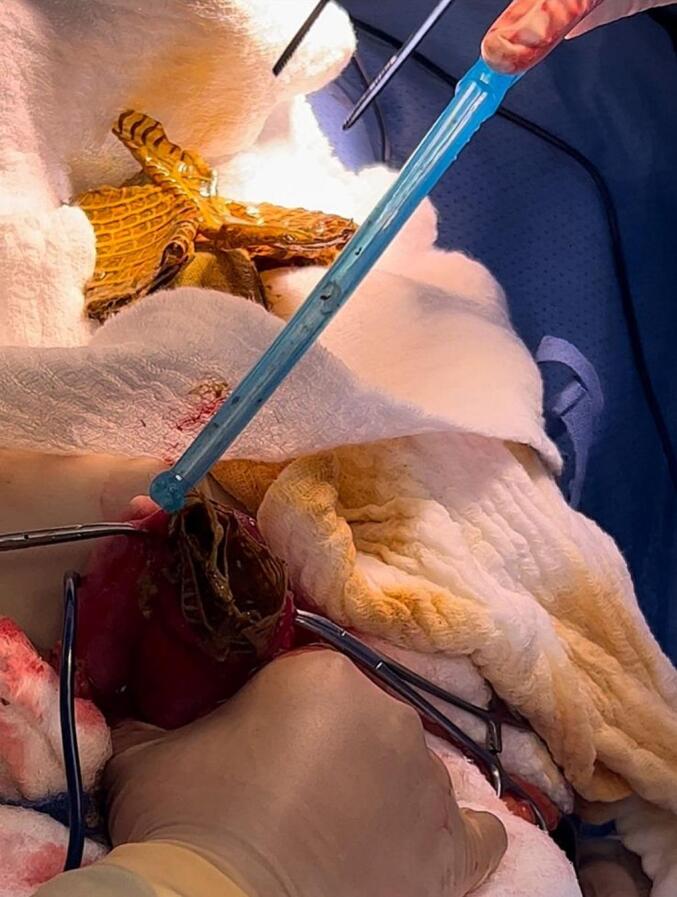
Extracted plastic table cover sheets.

The external findings on the exploratory laparotomy were that the small bowel was distended but viable with a collapsed cecum and colon. An intraluminal foreign body was identified 90 cm proximal to the ileocecal valve; the foreign body was approximately 13 cm in length and 4 cm in width; and another was noted in the stomach with approximately the same dimensions (Fig. 3, Fig. 4). The one in the small bowel was in the form of multiple folded sheets of plastic table cover that were extracted through a transverse enterotomy just proximal to the area of obstruction. Closure of the enterotomy was achieved in a hand sewn transverse fashion with two layers. The one in the stomach was similar and was extracted through an oblique gastrotomy incision on the anterior wall of the body of the stomach just proximal to the bezoar, and the gastrotomy incision was closed in the same fashion as the enterotomy. Examination of the rest of the bowel did not reveal any other lesions.

Postoperatively, the patient remained in the intensive care unit (ICU) due to difficult weaning from the ventilator and the presence of acute kidney injury requiring multiple renal replacement therapies. She was extubated successfully and transferred to the ward.

During her hospital stay, she was diagnosed with separation anxiety and was started on mirtazapine. Delightfully, the patient recovered well with no sequelae.

## 4 Discussion

The word bezoar originates from the Arabic word “Badzehr” or the Persian word “Panzer”, meaning antidote. In J.K. In Rowling's Harry Potter books, there was a reference to bezoars, and it was envisioned that these can protect from any toxins [2,3]. Bezoars rarely occur, but cases have been well documented [6,7]. The literature review revealed that most of the cases of ingestion of blunt foreign bodies occur in mentally challenged individuals, prisoners and those diagnosed with psychiatric illnesses [5,8,9,11]. The impaction of these materials often occurs in narrow areas such as the lower esophagus, duodenum, ileocecal valve or even the anus [8,10]. In this unique case, two points of impaction were noted: one in the small bowel and the other in the stomach. A total of approximately 10 folded sheets of plastic table cover were extracted. Although the patient's presentation was initially AKI and the diagnosis was established with no morbid complications, the emphasis on the importance of history taking when applicable must be considered a priority for early diagnosis and avoiding dreadful complications [5,8,10,11]. As a consequence, the physician must maintain a high index of suspicion when approaching such cases.

The diagnosis might be suspected based on the history and physical examination, but in this case, it was established by the CT KUB scan. The gold standard approach for foreign body diagnosis and management is endoscopy, in our patient the abdominal examination showed peritonitis which is most likely secondary to bowel obstruction that contributed to further bowel wall thickening and inflammation. Eventually, all of these changes can lead to bacteremia and peritonitis. Nonetheless, CT scan with contrast is a great tool and can be beneficial [8,10,11]. According to the literature, the management of foreign body ingestion can be different depending on the type of bezoar. An expectant management approach for blunt small objects could be attempted, as they can pass easily through the GIT with no complications in most cases. Endoscopic versus surgical or even chemical dissolution as a choice of treatment is dependent on multiple factors, such as the patient's status, available resources, foreign body characteristics and location. Therefore, the treatment plan is tailored to each patient individually [8,11]. The surgical approach can be open or laparoscopic, but the laparoscopic technique is favored, as it is minimally invasive with less postoperative pain and a quicker recovery, as in any laparoscopic surgery and it depends on the surgeon experience [6,10,11].

The outcome in these cases is generally favorable. Nevertheless, a preventive approach is better achieved to avoid morbidity and mortality at all costs. This can be achieved by increasing the awareness of high-risk patients and their relatives, as well as early psychiatric evaluation and dietitian assessment [11].

## 5 Conclusion

The bezoar is an uncommon entity that is underreported in the literature. As in our case, a bezoar was found simultaneously in the stomach and small bowel. The approach is multidisciplinary. Moreover, it depends on the availability of a qualified endoscopist, surgeon and psychiatrist. As the patient was diagnosed with a psychiatric illness during her hospital stay, mental health awareness was a concern and should be raised in the population via public health education and family awareness. Early identification of certain behaviors that might point to a psychiatric illness is crucial. Therefore, a psychiatric assessment is an important step in the management of these patients. Our approach was surgical, as the patient had developed signs of peritonitis. Therefore, early intervention in such a case can decrease the length of hospital stay and reduce complication rates.

## Abbreviations

GIT: Gastrointestinal tract
KSA: Kingdom of Saudi Arabia
SBO: Small bowel obstruction
AKI: Acute kidney injury
CT: Computerized tomography
KUB: Kidneys, ureters and bladder
NGT: Nasogastric tube
BUN: Blood urea nitrogen
ICU: Intensive care unit

## Consent

The patient's daughter gave verbal and written consent for publication.

## Ethical approval

Not applicable.

## Funding

None.

## Guarantor

Noran Sultan.

## Research registration number

Not applicable.

## CRediT authorship contribution statement

NS contributed to the literature review writing of the case, and HS and NS para-phrased and edited the case report. HA and HAL helped write the case report and edit the images.

## Declaration of competing interest

None.
